# Meta-analysis of the correlation between *Helicobacter pylori* infection and autoimmune thyroid diseases

**DOI:** 10.18632/oncotarget.22929

**Published:** 2017-12-04

**Authors:** Yi Hou, Wen Sun, Chengfei Zhang, Tieshan Wang, Xuan Guo, Lili Wu, Lingling Qin, Tonghua Liu

**Affiliations:** ^1^ Key Laboratory of Health Cultivation of the Ministry of Education, Beijing University of Chinese Medicine, Beijing, 100029, People’s Republic of China; ^2^ Dongfang Hospital of Beijing University of Chinese Medicine, Beijing, 100078, People’s Republic of China; ^3^ Beijing Institute of Chinese Medicine, Beijing University of Chinese Medicine, Beijing, 100029, People’s Republic of China; ^4^ Department of Science and Technology, Beijing University of Chinese Medicine, Beijing, 100029, People’s Republic of China

**Keywords:** helicobacter pylori infection, AITD, meta-analysis

## Abstract

**Objective:**

This study presents a systematic meta-analysis of the correlation between *Helicobacter pylori* (*H. pylori*) infection and autoimmune thyroid diseases (AITD).

**Materials and Methods:**

Fifteen articles including 3,046 cases were selected (1,716 observational and 1,330 control cases). These data were analyzed using Stata12.0 meta-analysis software.

**Results:**

H. pylori infection was positively correlated with the occurrence of AITD (OR = 2.25, 95% CI: 1.72–2.93). Infection with *H. pylori* strains positive for the cytotoxin-associated gene A (CagA) were positively correlated with AITD (OR = 1.99, 95% CI: 1.07–3.70). There was no significant difference between infections detected using enzyme-linked immunosorbent assay (ELISA) and other methods (χ2 = 2.151, *p* = 0.143). Patients with Grave’s disease (GD) and Hashimoto’s thyroiditis (HT) were more susceptible to *H. pylori* infection (GD: OR = 2.78, 95% CI: 1.68–4.61; HT: OR = 2.16, 95% CI: 1.44–3.23), while the rate of *H. pylori* infection did not differ between GD and HT (χ2 = 3.113, *p* = 0.078).

**Conclusions:**

*H. pylori* infection correlated with GD and HT, and the eradication of *H. pylori* infection could reduce thyroid autoantibodies.

## INTRODUCTION

Autoimmune thyroid diseases are familial autoimmune disorders that are more common in women than men. These diseases include Grave’s disease (GD), Hashimoto’s thyroiditis (HT), atrophic thyroiditis, and subacute lymphocytic thyroiditis (also known as postpartum thyroiditis, PPT), painless thyroiditis (PT), or silent thyroiditis (ST) [1]. The primary pathological features of autoimmune thyroid diseases are thyroid tissue infiltration of lymphocytes and thyroid dysfunction. Other typical hallmarks of these diseases are thyroid autoantibodies such as thyrotropin receptor antibody (TRAb), anti-thyroglobulin antibody (TGAb), and anti-thyroperoxidase antibody (TPOAb) [2]. The production of these autoantibodies can be attributed to both environmental and genetic factors [3]. Iodine overdose is the primary environmental cause [4], while bacterial and viral infections could be other causative factors [5, 6]. Cross-reactive antigens can induce superantigen-activated polyclonal T cells, increase expression of human leukocyte antigen in thyroid tissue and promote other autoimmune tolerance responses [7, 8]. *Helicobacter pylori (H. pylori)* infection is one of the most common chronic infections worldwide [9]. *H. pylori* specifically colonizes in the gastric mucosa, inducing chronic inflammation and promoting chronic gastritis, peptic ulcers, and gastric cancer [10]. *H. pylori* has been reported to be associated with other diseases such as diabetes [11], nonalcoholic fatty liver disease [12], iron deficiency anemia [13], and idiopathic thrombocytopenic purpura [14]. The relationship between *H. pylori* infection and AITD has also recently been explored. In 2013, Shi *et al.* [15] systematically evaluated 7 studies including 862 patients, observing that *H. pylori* infection was associated with the development of AITD in patients with GD but not HT. In this systematic meta-analysis, we further investigated the association between *H. pylori* infection and AITD.

## RESULTS

### Characterization of the included studies and method evaluation

**Figure 1:**
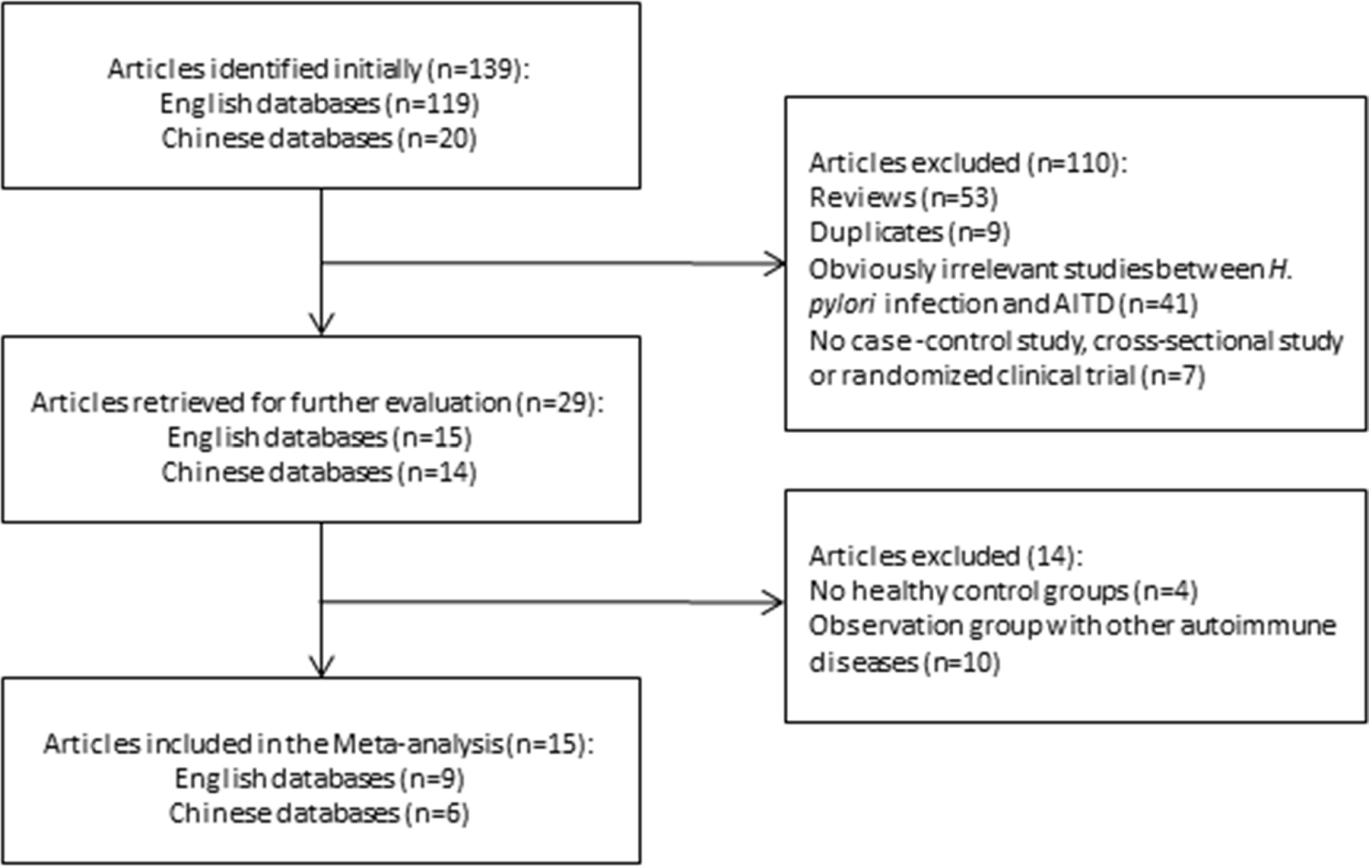
Flow chart of systematic literature review

**Figure 2:**
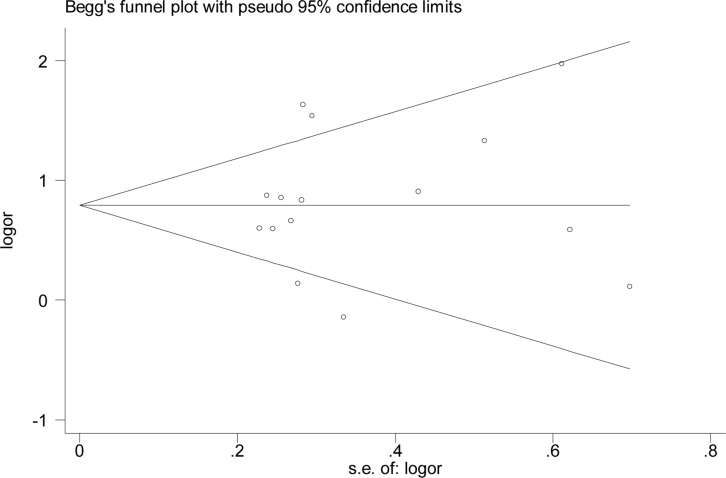
Funnel plot analysis of *H. pylori infection* and autoimmune thyroid diseases

The initial search identified 139 articles that investigated the association between H. pylori infection and AITD, including 119 articles in English and 20 in Chinese. Further examination excluded 110 articles, including 53 review articles, nine duplicated articles, 41 articles with unavailable information, and seven articles without controls, cohort, or cross-sectional surveys. The remaining 29 articles were carefully analyzed. Four articles had a control group that did not consist of healthy individuals, and ten articles with patients in the case group who had other autoimmune diseases, were excluded. Eventually, 15 articles [16–30] including 3,046 patients were selected for meta-analysis (Figure 1). The included studies included cases of GD and HT but no cases of atrophic thyroiditis or subacute lymphocyte thyroiditis. The identified articles were all case-control studies; including six published in Chinese [20, 23, 24, 27–29] and nine published in English [16–19, 21, 22, 25, 26, 30]. These studies included a total of 1,716 patients with AITD and 1,330 control subjects. Seven of these articles were conducted in China [20, 23, 24, 26–29], and eight were completed in other countries [16–19, 21, 22, 25, 30]. Of the 15 included articles, two were abstracts [16, 19] and 13 were full texts [17, 18, 20–30] (Table 1). All the articles had clear inclusion and exclusion criteria. The baseline characteristics of the participants in all the studies were comparable between the observation and control groups, apart from one study [30], in which the age of participants in the observation group was greater than the age of participants in the control group. One included study [18] was conducted on children with an average age of 10.6 years, while the other seven studies [16, 19, 20, 23, 24, 27, 29] reported no information on the age of the subjects. In nine studies [18–20, 22, 23, 25, 26, 29, 30] *H. pylori* infection was diagnosed by ELISA. Other methods, such as western blotting, urea breath test (UBT), stool antigen test (SAT), and both ELISA and UBT, were used in the remaining four studies [16, 17, 21, 24]. In two studies [27, 28], the diagnostic method was not reported. Overall, 11 studies [16, 18, 20, 21, 23–29] indicated that *H. pylori* infection was associated with AITD, while four studies [17, 19, 22, 30] showed no correlation between *H. pylori* infection and AITD. Seven of the 15 studies reported that patients were infected with CagA-positive strains of *H. pylori* [16, 17, 19, 21, 22, 26, 30], of which four studies [16, 21, 22, 26] showed that the infection was significantly associated with AITD, and three studies demonstrated no correlation between the infection and AITD [17, 19, 30]. Five studies reported that thyroid autoantibody levels in patients in the observation group were influenced by eradication therapy [20, 24, 27–29]. The logarithm and logarithm standard error of OR value in *H. pylori* infection were utilized to draw the funnel plot, which was symmetrical and was analyzed using Begg’s rank correlation method, with Pr > |z| = 0.656 > 0.05, suggesting the absence of publication bias (Figure 2).

**Table 1:** Characterization of included studies and method evaluation

Researcher and date	Nation	Study type	Age (years)		Sex (F/M)	H.pylori test method	AITD		control		CagA positive AITD/ control	NOS Quality Score
			AITD	control			Hp-Po	n	Hp-Po	n		
Figura N, et al. 1999 [16]	Italy	Case-control study	NR	NR	All women	WB	32	41	16	33	23/8	unclear
Franceschi F, et al. 2004 [17]	Italy	Case-control study	43.6 ± 11	44.2 ± 12	32/4	UBT	6	16	7	20	3/3	4
Larizza D, et al. 2006 [18]	Italy	Case-control study	11.2	10	130/30	Elisa	24	90	9	70	NR	6
Sterzl I, et al. 2006 [19]	Czech	Case-control study	NR	NR	NR	Elisa	13	60	4	30	5/6	unclear
Chen LM, et al. 2012 [20]	China	Case-control study	35.83 ± 12.12	NR	NR	Elisa	166	246	64	120	NR	8
Bassi V, et al. 2012 [21]	Italy	Case-control study	49.2 ± 6.9	49.0 ± 4.5	192/20	SAT	71	112	43	100	61/21	6
Soveid M, et al. 2012 [22]	Iran	Case-control study	35.9 ± 8.6	37.4 ± 6.1	159/41	Elisa	66	88	87	112	43/28	6
Gao Y, et al. 2013 [23 ]	China	Case-control study	NR	NR	168/58	Elisa	98	126	43	100	NR	7
Chen LM, et al. 2013 [24]	China	Case-control study	33.5 ± 8.9	NR	NR	Elisa, UBT	84	122	64	120	NR	8
Aghili R, et al. 2013 [25]	Iran	Case-control study	34.4 ± 11.1	34.8 ± 7.8	66/14	Elisa	20	43	4	37	NR	6
Wang Y, et al. 2013 [26]	China	Case-control study	28 ± 5.4	26.9 ± 5.5	181/137	Elisa	143	216	53	102	75/15	7
Zhao YH, et al. 2014 [27]	China	Case-control study	45.9	NR	NR	NR	96	150	64	150	NR	5
Fei AW, et al. 2014 [28]	China	Case-control study	40.8 ± 6.5	40.5 ± 6.6	212/148	NR	120	180	46	100	NR	6
Wang MR, et al. 2015 [29]	China	Case-control study	35–55	NR	NR	Elisa	98	125	52	125	NR	7
Shmuely H, et al. 2016 [30]	Israel	Case-control study	48.3 ± 16.6	43.5 ± 16.6	All women	Elisa	47	101	48	111	10/15	7

Note: AITD, autoimmune thyroid diseases; Elisa, enzyme-linked immunosorbent assay; F, female; *Hp*-Po, *Helicobacter pylori* positive; M, male; NR, not reported; SAT, stool antigen test; UBT, urea breath test; WB, western blotting.

### Meta-analysis results

#### Comparison of *H. pylori* infection rates

**Figure 3:**
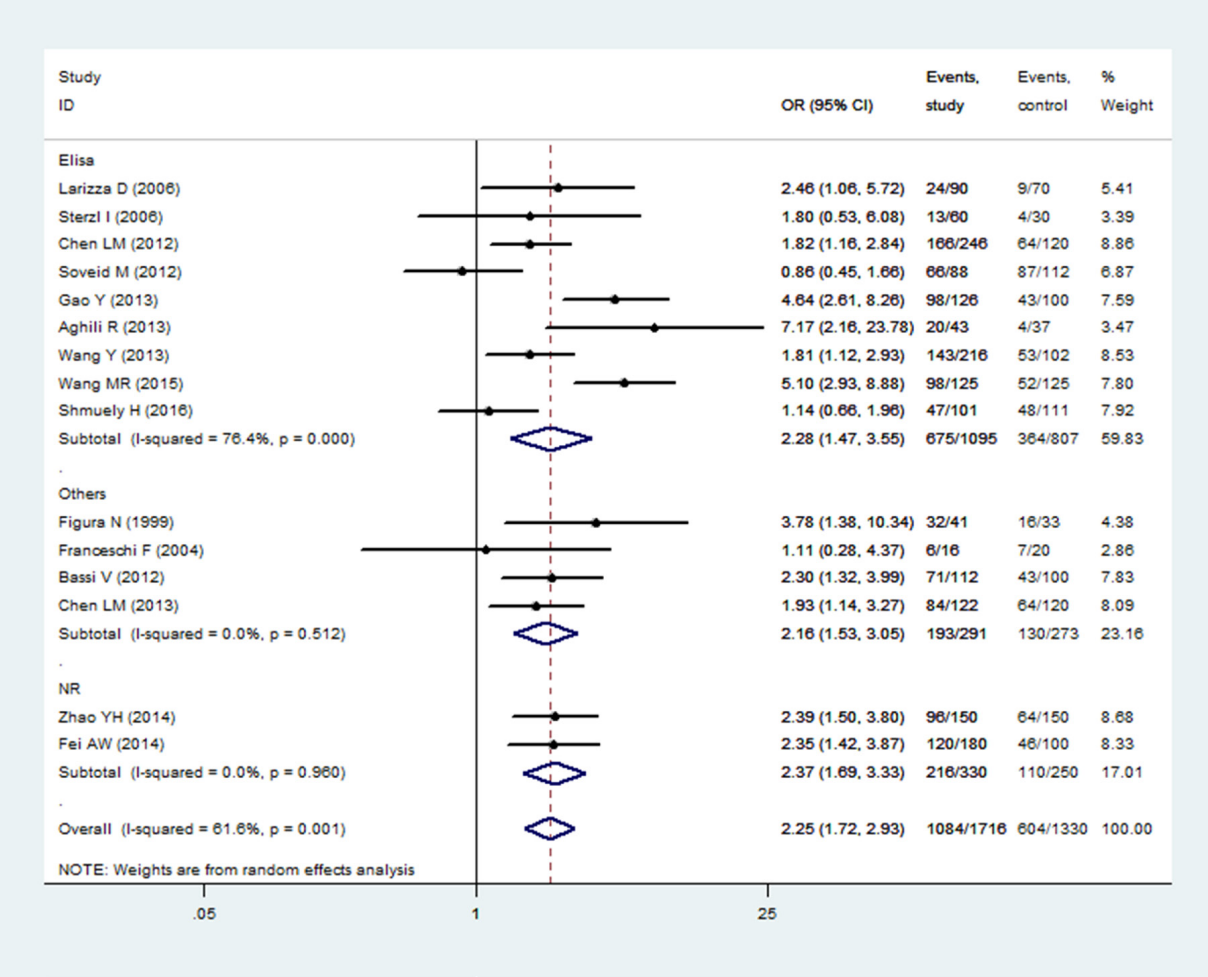
Correlation analysis of *H. pylori infection* with AITD by different detection methods Note: AITD, autoimmune thyroid diseases; Elisa, enzyme-linked immunosorbent assay; Others, other methods for detecting *H. pylori* infection; NR, no reported.

The *H. pylori* infection rate was reported in 15 studies. As there was heterogeneity among the studies (I^2^ = 61.6%, *p* = 0.001), a random model was established. The *H. pylori* infection rates among participants in the observation and healthy control groups were 63.17% and 45.41% respectively, indicating that *H. pylori* infection was correlated with AITD (OR = 2.25, 95% CI: 1.72–2.93, *p* < 0.001). The relationship between *H. pylori* infection diagnostic method was further investigated by subgroup analysis. In participants diagnosed using ELISA, the infection rate was 61.64% in the observation group and 45.11% in the control group (OR = 2.28, 95 % CI: 1.47–3.55, *p* < 0.001). Participants diagnosed using other diagnostic methods had an infection rate of 66.32% in the observation group and 47.62% in the control group (OR = 2.16, 95% CI: 1.53–3.05, *p* < 0.001). Both subgroup analyses indicated that the infection rate was higher in the observation group than in the control group (Figure 3). There was no significant difference in the rate of diagnosis via ELISA and other methods according to the χ^2^ test (χ^2^ = 2.151, *p* = 0.143).

**Figure 4:**
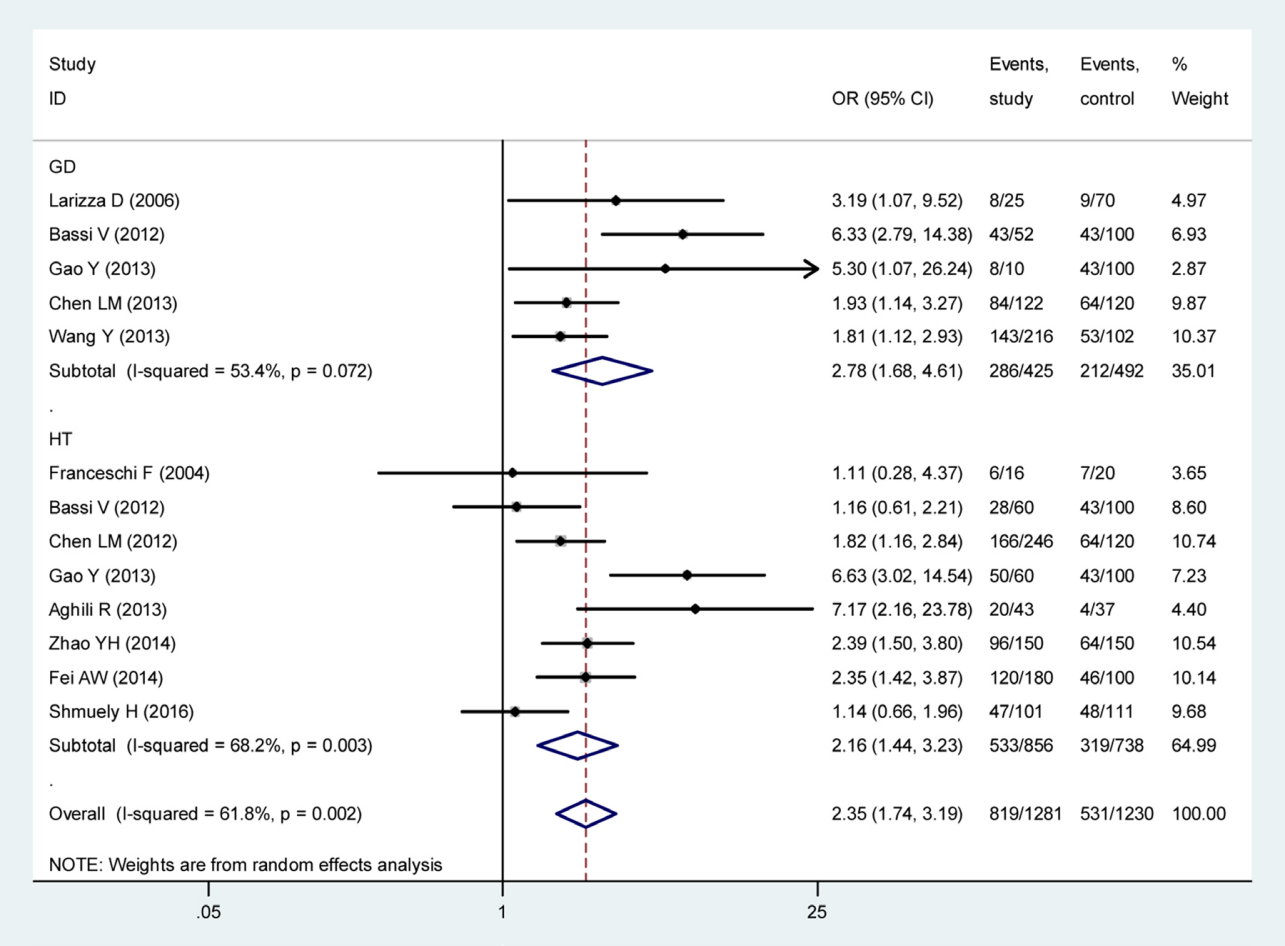
Correlation analysis of *H. pylori* infection with GD and HT Note: GD, Grave’s disease; HT, Hashimoto’s thyroiditis.

Further subgroup analysis revealed that the rate of *H. pylori* infection differed significantly between the GD group (67.29%) and matched controls (43.09%, OR = 2.78, 95% CI: 1.68–4.61, *p* < 0.001) and the HT group (62.27%) and matched controls (43.22%, OR = 2.16, 95% CI: 1.44–3.23, *p* < 0.001, Figure 4). The rate of *H. pylori* infection was not significantly different between participants with GD and HT (χ^2^ = 3.113, *p* = 0.078).

Due to the observed heterogeneity in statistical results, a sensitivity analysis was performed to investigate the influence of specific research methods. This analysis indicated no significant difference and the conclusions were consistent, indicating that the analysis was stable (Supplementary Figure 1). Meta-regression analysis was performed to determine the heterogeneity in the results, and revealed no heterogeneities among the year and country of publication, sample size, case comparison ratio, or the method used to detect the presence of *H. pylori* (Supplementary Table 1).

### Rates of infection with CagA-positive *H. pylori* strains

**Figure 5:**
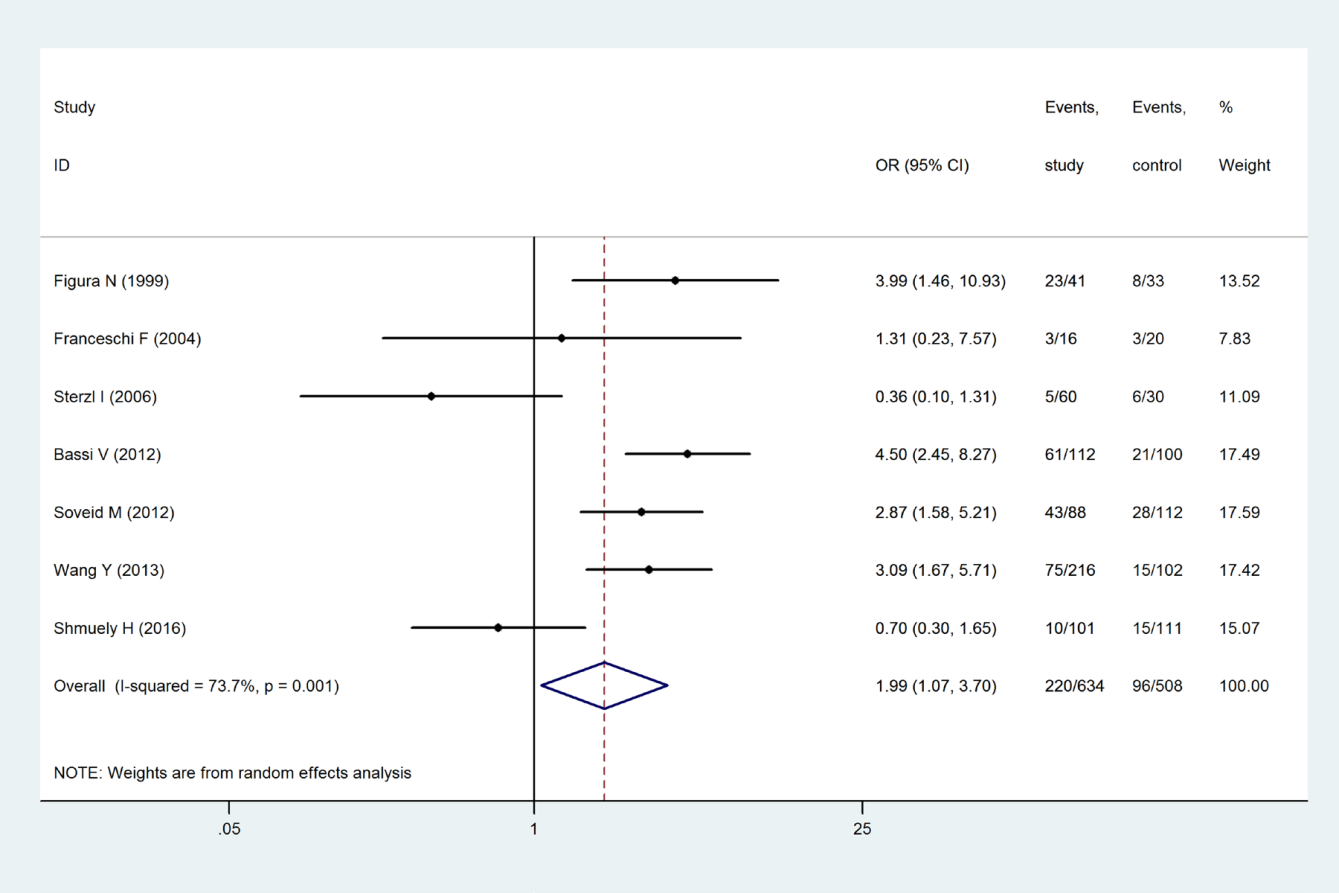
Correlation analysis of infection with a CagA-positive strain of *H. pylori*, and AITD

The rates of infection with CagA-positive *H. pylori* strains were reported in seven studies. Heterogeneity was observed among the rates in these studies (I^2^ = 73.7%, *p* = 0.001). According to random model calculation, the rate of *H. pylori* infection was 34.70% in the observation group and 18.90% in the control group (OR = 1.99, 95% CI: 1.07–3.70, *p* = 0.030), indicating that infection with CagA-positive *H. pylori* strains was associated with AITD (Figure 5). Consistent with this analysis, there was heterogeneity in the statistical results and sensitivity analysis. Systematic evaluation was carried out in a manner similar to that described previously. By excluding the studies by Sterzl *et al.* [19] and Shumuely *et al.* [30], the merged effect significantly changed (OR = 3.35, 95% CI: 2.42–4.64) and the heterogeneity disappeared (I^2^ = 0%, *p = 0.653*), while the conclusion was still consistent (Supplementary Figure 2). Due to the limitations of the retrieved literature, meta-regression analysis was unable to identify the source of heterogeneity [31, 32].

### Eradication therapy for thyroid autoantibody levels

Five studies [20, 24, 27–29] reported the influence of eradication therapy on thyroid autoantibodies. Patients with AITD who had *H. pylori* infection were selected from each study and were randomly allocated to observation and control groups. Only patients in the observation group were treated with eradication therapy. Therefore, meta-analysis could not be performed based on the data provided. The descriptive analysis is shown in Supplementary Table 2.

## DISCUSSION

The correlation between AITD and *H. pylori* infection was studied in 15 publications reporting a total of 3,046 cases. Unlike a previous meta-analysis published by Shi *et al.* in 2013 [15], which included unhealthy individuals in the control group, and a previous meta-analysis by Luis *et al.* in 1998 [33], which included patients with non-toxic nodular goiter, in this study we included only healthy individuals in the control group. Thyroid nodules were previously reported to be associated with *H. pylori* infection [34]. Atrophic gastritis and/or chronic *H. pylori* infection in patients with multiple nodular goiters are often treated with an increased dose of thyroxine [35], hence, we excluded studies performed on such patients.

Our results indicated that patients with AITD were more susceptible to *H. pylori* infection (OR = 2.25, 95% CI: 1.72–2.93), particularly infection with CagA-positive strains of *H. pylori* (OR = 1.99, 95% CI: 1.07–3.70). This analysis is consistent with a previous report by Shi *et al.* (OR = 1.92, 95% CI: 1.41–2.61; OR = 2.24, 95% CI: 1.06–4.75]. The CagA gene is present in high-toxicity strains of *H. pylori* and the encoded product, CagA protein, is a marker of gastric mucosal inflammation. CagA is highly immunogenic and promotes local inflammatory cell infiltration [36]. CagA-positive *H. pylori* strains share a highly identical sequence with thyroperoxidase [37], hence, *H. pylori* infection could induce autoantibody damage to gastric epithelial cells, leading to gastric disease and antigenic antibody cross-reactions causing thyroid tissue damage [38, 39]. Shi *et al.* indicated that *H. pylori* infection was associated with GD (OR = 4.35, 95% CI: 2.48–7.64), but showed no significant correlation with HT (OR = 1.45, 95% CI: 0.92–2.26). Both GD and HT were included in this study and were associated with *H. pylori* infection in subgroup analysis (OR = 2.78, 95% CI: 1.68–4.61; OR = 2.16, 95% CI: 1.44–3.23). GD and HT share similar pathogenic factors, pathology, biochemistry, and clinical features such as thyroid tissue lymphocyte infiltration resulting in inflammation and TPOAb, TGAb, or increased levels of other thyroid autoantibodies [40]. Thus, *H. pylori* infection is expected to be associated with GD and HT, which is consistent with the results of our meta-analysis. We also observed that pharmaceutical eradication of *H. pylori* infection reduced levels of thyroid autoantibodies in patients with GD and HT [41]. Patients with AITD who had dysfunctional gastric acid secretion required treatment with higher thyroid hormone levels, indicating that normal gastric acid secretion was necessary for the effective absorption of oral thyroxine [35]. These studies suggest that *H. pylori* infection was associated with the pathogenesis and development of AITD. Our literature review identified no articles reporting the association between atrophic thyroiditis and *H. pylori* infection, while the conclusions from other studies are controversial. Due to the limited number of publications and limited data, the correlation between atrophic thyroiditis and *H. pylori* infection was not analyzed here.

Shi *et al.* previously reported that *H. pylori* infection diagnosed by ELISA was not associated with AITD. However, we found that regardless of diagnostic test, *H. pylori* infection was robustly associated with AITD. Although there was great heterogeneity in the merged effects, meta-regression analysis showed that the varieties of diagnostic tools were not responsible for this heterogeneity. Shi *et al.* attributed the lack of relationship between ELISA-diagnosed *H. pylori* infection and AITD to the inability of this assay to distinguish active from resolved infection, and thus included participants with resolved infection. However, most of the studies that we included did not report a history of AITD. As AITD is a chronic process, *H. pylori* infection may occur at any stage, which could be either the cause or the consequence of patients susceptible to the infection. Shi et al.’s and our meta-analysis evaluated only whether *H. pylori* infection was associated with AITD without considering whether active infection had any effect on the results.

In conclusion, *H. pylori* infection is associated with AITD. Our results suggest that patients with AITD are more susceptible to *H. pylori* infection, especially infection with CagA-positive strains of *H*. *pylori*. The high heterogeneity could be influenced by several factors. First, only the abstracts of some included publications were available in standard databases such as PubMed, Web of Science, Ovid Online, etc. [16, 19], limiting the extraction of patient information. In addition, *H. pylori* infection could also be associated with other non-gastrointestinal diseases, such as diabetes or autoimmune diseases, but we did not exclude these cases from our analyses [18, 22, 25, 30]. Additionally, in some studies antibiotic use was not ruled out [18, 26]. These three factors may contribute to the heterogeneity of these studies. Additionally, although the funnel plot suggests no bias, we cannot ignore the potential language or regional bias as we included only manuscripts published in English and Chinese.

## MATERIALS AND METHODS

### Inclusion criteria

Articles or publications including the following information were selected:

(1) Case-control, cohort, or cross-sectional studies in which the number of individuals in the observation group, control group, and number of individuals infected with *H. pylori*, were in either Chinese or English in the abstract or full-text of the publication. (2) The observation groups were diagnosed with AITD. GD was diagnosed according to the following criteria: hyperthyroidism, decreased TSH, increased FT3 and FT4, diffuse enlargement of thyroid tissue, and antibodies against TSH receptor (TRAb) and/or TPOAb- and TGAb-positivity. HT was diagnosed according to the following criteria: hypothyroidism, increased TSH, decreased FT3, FT4, TPOAb- and TGAb-positivity, typical ultrasonographic features, and evaluation by fine needle aspiration thyroid cytology tests. Atrophic thyroiditis was diagnosed according to the following criteria: hypothyroidism, increased thyroid autoantibodies, and thyroid atrophy features by ultrasound evaluation. PPT was diagnosed according to the following criteria: normal thyroid function before or during pregnancy, abnormal thyroid function within one year after childbirth or abortion, no thyroid pain, low rate of iodine uptake and high blood TPOAb levels, and ultrasound examination revealing diffuse or nodular swelling of the thyroid gland. PT or ST were diagnosed according to the following criteria: sudden onset hyperthyroidism without pain, low iodine uptake rate and high blood TPOAb, with remission or development of permanent hypothyroidism in 6–12 months, and an ultrasound examination revealing diffuse swelling of the thyroid gland. Control groups included healthy individuals without any autoimmune disease. (3) *H. pylori* infection was determined by at least one of the following diagnostic methods: stool antigen test, urea breath test, ELISA, or Western blotting.

### Exclusion criteria

Articles or publications were excluded where (1) Insufficient information was provided to support the association of *H. pylori* infection with AITD in the full text publication or abstract where full text manuscripts were not available; (2) The same data was reported in several publications; (3) The study did not include a control group.

### Search strategy

The following databases were searched for articles published before July 2017: PubMed, Medline, Web of Science, OVID, SINOMED, VIP Chinese Science and Technology Journal Full-text Database, Wanfang Data Resources, and CNKI full-text database. English and Chinese search terms included *Helicobacter pylori*, *H. pylori*, autoimmune thyroid disease, autoimmune thyroiditis, lymphocytic thyroiditis, Grave’s disease, Hashimoto’s thyroiditis, atrophic thyroiditis, postpartum thyroiditis, painless thyroiditis, and silent thyroiditis. Where the same data was analyzed by multiple studies, the most recent results were used.

### Quality assessment and data extraction

The quality of the retrieved articles was evaluated independently by two researchers. In case of any disagreement between the researchers, the final decision was made by a third researcher. The quality assessment was followed by the standard “NOS” evaluation recommended by Cochrane [42], including 3 items and 8 articles (population selection, comparability, exposure evaluation, and outcome evaluation) with a maximum score of nine. The results included diagnostic criteria, the case number of both *H. pylori* and CagA-positive infections, the sample numbers in observation and control groups, and autoantibody titer after pharmaceutical eradication of *H*. *pylori* infection.

### Statistical process

Heterogeneity analysis was performed using Stata12.0 processing software. If heterogeneity (I^2^ > 50%, *p* < 0.10) was found between studies, a random model was used for further calculation. In the case of no heterogeneity, the fixed effects model was used. The results were presented as the odds ratio (OR) and confidence interval (95% CI). Meta-regression analysis was used to identify heterogeneity sources. The stability of the meta-analysis was assessed using sensitivity analysis. If the data provided could not be meta-analyzed, only qualitative analysis by description was provided. An χ2 test was performed using SPSS16.0 to assess differences between subgroups. The funnel plot and Begg’s rank correlation method were used to identify biases in the data.

## SUPPLEMENTARY MATERIALS FIGURES AND TABLES



## Abbreviations

AITD: autoimmune thyroid diseases
CagA: cytotoxin-associated gene A
CI: confidence interval
ELISA: enzyme-linked immunosorbent assay
GD: Grave’s disease
*H. pylori*: *Helicobacter pylori*
HT: Hashimoto’s thyroiditis
NR: not reported
OR: odds ratio
PPT: postpartum thyroiditis
PT: painless thyroiditis
ST: silent thyroiditis
SAT: stool antigen test
TGAb: anti-thyroglobulin antibody
TPOAb: anti-thyroperoxidase antibody
TRAb: thyrotropin receptor antibody
UBT: urea breath test
WB: western blotting
