# Supplementary material for: Meta-analysis of the correlation between Helicobacter pylori infection and autoimmune thyroid diseases
Source: Oncotarget. 2017 Dec 4;8(70):115691–700. doi: 10.18632/oncotarget.22929 (PMC5777804; doi:10.18632/oncotarget.22929)
Supplement: Supplementary file 1 [file oncotarget-08-115691-s001.pdf]

Meta-analysis of the correlation between *Helicobacter pylori* infection and autoimmune thyroid diseases

SUPPLEMENTARY MATERIALS

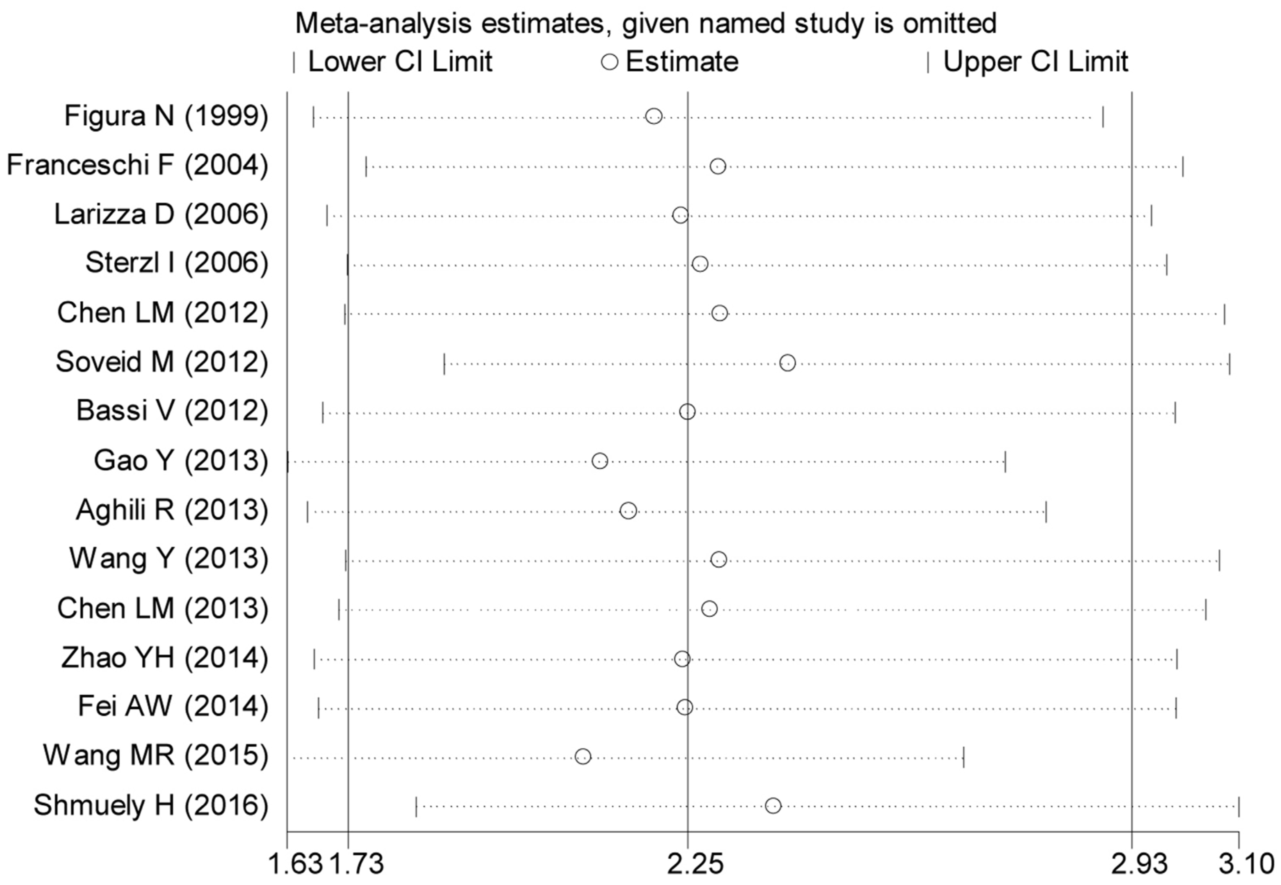

Supplementary Figure 1: Sensitivity analysis of the correlation between *H. pylori* infection and AITD.

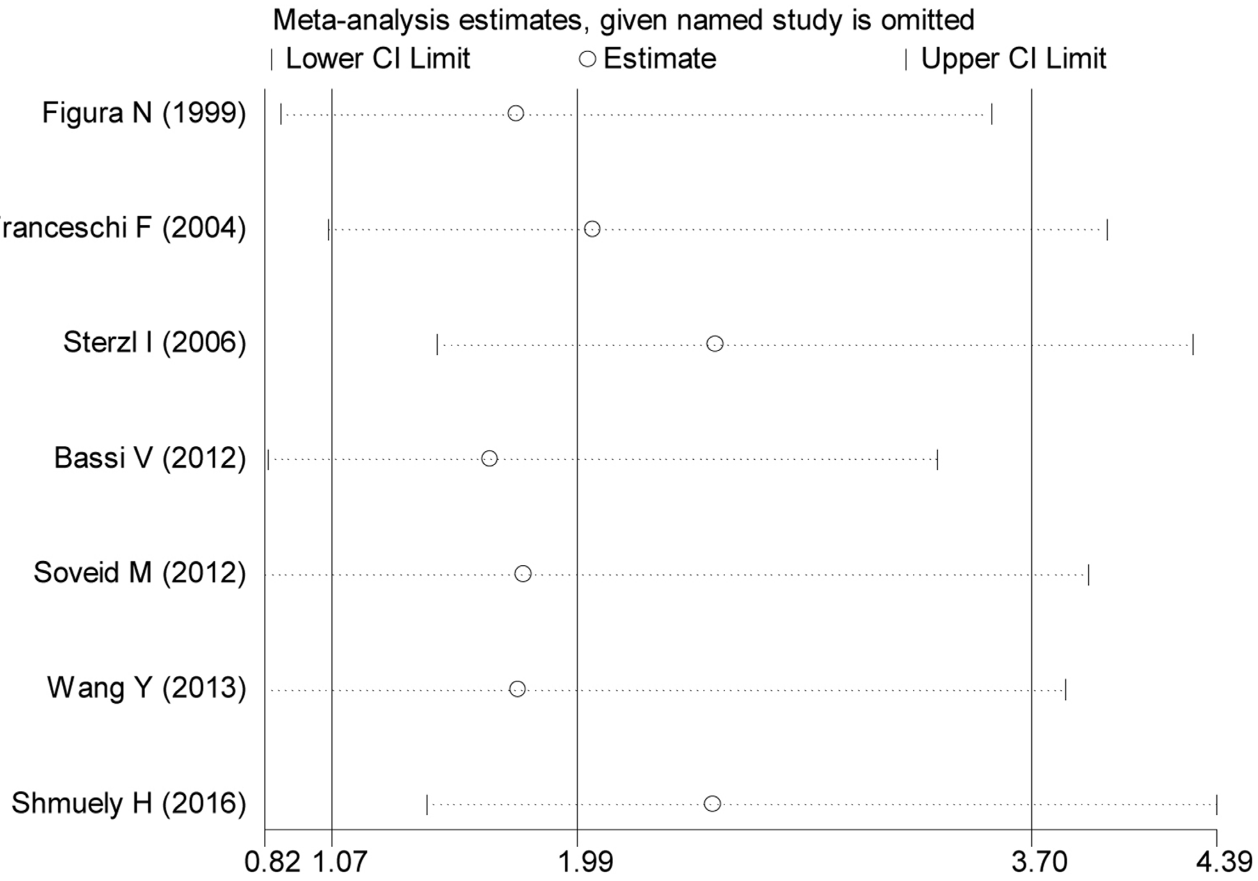

Supplementary Figure 2: Sensitivity analysis of the correlation between infection with CagA-positive strain of *H. pylori* and AITD.

Supplementary Table 1: Meta-regression analysis of the correlation heterogeneity of *H. pylori* infection and autoimmune thyroid diseases

logor	Coef	Std. Err	*t*	*p* > | t |	[95% Conf.	Interval]
Date of publication	.0802023	.0782668	1.02	0.332	−.0968495	.2572541
Nation	−.2484785	.2053655	−1.21	0.257	−.7130476	.2160905
Sample size	−.0031961	.0032243	−0.99	0.347	−.01049	.0040978
Case–control ratio	.1507225	.4557598	0.33	0.748	−.8802779	1.181723
H. pylori infection test method	.2291451	.2628453	0.87	0.406	−.3654523	.8237424
_cons	−159.7303	156.7992	−1.02	0.335	−514.4347	194.974

Supplemental Table 2: Analysis of thyroid autoantibody titer after *H. pylori* infection eradication

Publication	AITD with eradication therapy (*n*)		Results	
	observation group	control group	Statistics	Clinical significance
Chen LM, et al. 2012 [20]	83	83	observation group: TPOAb: Before treatment 257.00 (88.86–1549.20),After treatment 103.00 (19.40–386.29), $p < 0.01$; TGAb: before treatment 592.50 (155.13–3784.50), after treatment 203.00 (19.30–1183.00), $p < 0.01$. control group: TPOAb: before treatment 249.00 (80.44–1803.11), after treatment 246.70 (46.34–1728.30), $p > 0.05$; TGAb: before treatment 573.00 (134.39–3743.9), after treatment 532.00 (67.19–3581.50), $p > 0.05$.	Antibody levels differed significantly before and after eradication therapy in the observation group, but did not differ in the control group, suggesting that H. pylori infection was associated with the development of Hashimoto's thyroiditis.
Zhao YH, et al. 2014 [27]	48	48	observation group: TPOAb: before treatment 267.3, after treatment 105.4, $p < 0.05$; TGAb: before treatment 586.3, after treatment 220.8, $p < 0.05$. control group: TPOAb: before treatment 253.4, after treatment 249.7, $p > 0.05$; TGAb: before treatment 577.6, after treatment 553.1, $p > 0.05$.	As above.
Fei AW, et al. 2014 [28]	60	60	observation group: TPOAb: before treatment 256.6 ± 45.2, after treatment 101.4 ± 21.4, $p > 0.01$; TGAb: before treatment 587.5 ± 78.4, after treatment 202.4 ± 41.4, $p > 0.01$. control group: TPOAb: before treatment 250.3 ± 41.7, after treatment 248.5 ± 40.2, p > 0.05; TGAb: before treatment 577.3 ± 72.6, after treatment 568.5 ± 77.3, $p > 0.05$.	As above.
Wang MR, et al. 2015 [29]	50	50	observation group: TPOAb: before treatment 261.00, after treatment 101.20, $p < 0.05$; TGAb: before treatment 549.78, after treatment 201.00, $p < 0.05$. control group: TPOAb: before treatment 251.00, after treatment 246.00, $p > 0.05$; TGAb: before treatment 554.00, after treatment 529.00, $p > 0.05$.	Antibody levels differed significantly before and after eradication therapy in the observation group, but not in the control group, suggesting that H. pylori infection was associated with the development of AITD.
Chen LM, et al. 2013 [24]	42	42	observation group: TRAb: before treatment 15.7 ± 4.6, after treatment decreasing level: 12.4 ± 4.1, $p > 0.05$; control group: before treatment 15.8 ± 4.9, after treatment decreasing level: 12.9 ± 4.6, $p > 0.05$. Remission rate of hyperthyroidism: observation group: 71.4%, control group: 47.6%, $p < 0.05$.	TRAb levels did not differ significantly before and after eradication therapy in the observation group. The rate of hyperthyroidism remission in the observation group was significantly higher than in the control group, suggesting that H. pylori infection was associated with Grave's disease.
